# Monthly versus daily administration of vitamin D3 in children: a retrospective propensity score-matched study

**DOI:** 10.3389/fendo.2023.1265943

**Published:** 2023-10-24

**Authors:** Jinjoo Choi, Yunsoo Choe, Seung Yang

**Affiliations:** ^1^ Department of Pediatrics, Hanyang University Seoul Hospital, Seoul, Republic of Korea; ^2^ Department of Pediatrics, Hanyang University Guri Hospital, Guri, Republic of Korea; ^3^ Department of Pediatrics, College of Medicine, Hanyang University, Seoul, Republic of Korea

**Keywords:** vitamin D deficiency, vitamin D, children, adolescent, administration and dosage, treatment outcome, safety, retrospective studies

## Abstract

**Objectives:**

We aimed to evaluate the efficacy and safety of monthly vitamin D3 administration compared to a daily dosing regimen in healthy children with vitamin D deficiency.

**Methods:**

This retrospective study included vitamin D deficient (serum 25-hydroxyvitamin D [25(OH)D] < 20 ng/mL) children with precocious puberty who received gonadotropin-releasing hormone agonist every 4 weeks between December 2019 and November 2022. We used propensity scores to 1:1 match daily (1,000 IU daily) and monthly (25,000 IU per 4 weeks) administration of vitamin D3 based on age, sex, body mass index Z-scores, season of blood collection, and baseline serum 25(OH)D concentrations.

**Results:**

Of 299 children, 192 were matched based on propensity scores (126 girls and 66 boys, 10.5 ± 1.4years). After a mean follow-up of 5.9 months (standard deviation [SD] 2.5 months), the monthly group showed a statistically significant increase in serum 25(OH)D concentrations (10.9 ± 5.3 vs. 8.2 ± 7.2 ng/mL; p = 0.018), higher corrected dose-response (12.3 ± 5.9 vs. 8.2 ± 7.2 ng/mL increase per 1,000 IU daily; p = 0.002), and a higher proportion of patients attaining 25(OH)D > 20 ng/mL (78.1% vs. 58.3%,; p=0.005) compared with the daily group. No cases of hypercalcemia were observed in either group.

**Conclusions:**

Monthly administration of vitamin D3 may be an effective and safe alternative to correct hypovitaminosis D in pediatric population, possibly attributed to enhanced compliance.

## 1 Introduction

Vitamin D plays important roles in the promotion of musculoskeletal health, maintenance of the immune system, glucose and insulin metabolism, and the cardiovascular system (1). Vitamin D is mainly synthesized upon exposure to ultraviolet B (UVB) radiation in sunshine (2). However, the contemporary lifestyle, including sedentary habits and increased indoor activities, leads to insufficient outdoor playtime in children, which results in the prevalence of hypovitaminosis D (3). South Korea is located in the Northern hemisphere (33-38°N), where limited amounts of UVB reach the ground during winter and spring. Consequently, synthesizing a sufficient amount of vitamin D from sunlight is challenging (4). According to a report from the Korean National Health and Nutrition Examination Survey, vitamin D deficiency is common and showed a significant increase in prevalence in Korea, rising from 52% to 75% in males and 68% to 83% in females between 2008 and 2014 (5).

Various guidelines have proposed adequate dietary reference intakes (DRIs) of vitamin D3. The American Academy of Pediatrics (6) recommended 600 IU daily intake to maintain 25-hydroxyvitamin D [25(OH)D] concentrations > 20 ng/mL, and the Endocrine Society (7) recommended a daily intake of 600 - 1000 IU to maintain serum 25(OH)D concentrations > 30 ng/ml in children aged 1–18 years. However, in clinical practice, children and adolescents often experience difficulties maintaining long-term adherence to daily dosing owing to low compliance (8). Thus, weekly or monthly administration of vitamin D3 has been attempted by some researchers and has shown somewhat favorable results (9–12), although consensus regarding the optimal interval and dose has not been reached. The long half-life of vitamin D3 supports a preference for intermittent regimens, which can potentially reduce the costs associated with non-adherence.

In this study, we aimed to compare the efficacy and safety of monthly vitamin D3 administration in achieving serum 25(OH)D concentrations higher than 20 ng/ml with those of a conventional daily regimen in children. We retrospectively reviewed children who were diagnosed with precocious or advanced puberty and received a gonadotropin-releasing hormone (GnRH) agonist to minimize the confounding effect of puberty on serum 25(OH)D level (13) and to ensure compliance by prescribing vitamin D3 at the same time as the GnRH agonist injection. We also compared the effects of increasing the 25(OH)D concentrations with the same cumulative daily dose of vitamin D3.

## 2 Materials and methods

### 2.1 Study participants

This retrospective cohort study included children who visited the Division of Pediatric Endocrinology between December 2019 and November 2022 at Hanyang University Hospital, Seoul and Guri. Each hospital is located in Seoul and Gyeonggi-do at a latitude of 37.5 ° N. All participants included in this study were administered GnRH agonists every 4 weeks after the diagnosis of precocious or advanced puberty. Among the 338 patients initially included, we excluded: (1) children with underlying diseases or conditions affecting vitamin D and bone metabolism such as endocrine disorders (growth hormone deficiency, diabetes mellitus, and thyroid disease) and severe illnesses affecting general nutritional status such as hemato-oncologic, neurological, hepatic, and chronic kidney diseases; (2) children taking any calcium or vitamin D supplementation at the time of the initial assessment; and (3) children with serum 25(OH)D concentrations above 20 ng/ml. The patients started taking vitamin D3 daily or monthly. According to the Endocrine Society guidelines (7), patients in the daily group were given either 600 IU or 1000 IU of oral vitamin D3 supplement. However, only patients taking 1000 IU were included in this study to allow for comparison with monthly administration. A daily dose of 1,000 IU was administered as five droplets of liquid, with each droplet containing 200 IU (100,000 IU/10 mL, Abiogen Pharma S.p.A.), whereas a dose of 25,000 IU was administered as 2.5 mL (25,000 IU/2.5 ml, Abiogen Pharma S.p.A.) once every 4 weeks. Vitamin D3 content of liquids was analyzed by the Ministry of Food and Drug Safety of the Republic of Korea to contain cholecalciferol within 90-105% (9,000 ~10,500IU/mL) of the labeled amount. To account for seasonal variability, patients were grouped according to the season of the first assessment: June to November as the summer/fall group and December to May as the winter/spring group.

The study protocol was approved by the Institutional Review Board of Hanyang University Hospital (IRB No. HYUH 2023-04-004). The requirement for informed consent was waived due to the retrospective nature of the study.

### 2.2 Biochemical assessments and anthropometric measurements

All patients underwent two consecutive clinical and biochemical evaluations at baseline and after vitamin D3 supplementation. The serum concentrations of total calcium and 25(OH)D were measured at each visit. Height (cm) was measured to the nearest 0.1 cm using a Harpendon stadiometer (Holtain Ltd., Crymych, Wales, UK), and weight (kg) was measured to the nearest 0.1 kg using a digital scale. Body mass index (BMI) was calculated as weight (kg) divided by the height (m) squared and expressed as kg/m^2^. Age- and sex-specific Z-scores for height, weight, and BMI were assessed based on the 2017 Korea National Growth Charts (14). Vitamin D deficiency was defined as a serum 25(OH)D level < 20 ng/mL (7).

### 2.3 Statistical analysis

Continuous data are presented as mean ± standard deviation, and the differences between the two groups were compared using Student’s t-test. Categorical variables were expressed as numbers (percentages), and comparisons between two groups were analyzed using the chi-square or Fisher’s exact tests. All statistical analyses were performed using R version 4.2.3 software (R Foundation for Statistical Computing, Vienna, Austria). Statistical significance was set at P < 0.05.

Owing to the retrospective nature of this study, there may have been confounding bias in selecting the method of vitamin D3 administration. Additionally, BMI, the season of blood collection, baseline 25(OH)D concentrations may have affected follow-up 25(OH)D concentrations. Hence, we performed propensity score matching to ensure homogeneity between the groups using the nearest neighbor matching method. A multivariate logistic regression model was constructed to predict the propensity score, which included age, sex, BMI Z-score, season of blood collection (summer/fall and winter/spring), and baseline 25(OH)D concentrations. Subsequently, patients were matched in a 1:1 ratio with a caliper of 0.3 times the standard deviation of the logit propensity score. Matching was performed using the MatchIt function in R. Standardized mean difference (SMD) was calculated to assess the balance of variables between daily and monthly groups, and SMD less than 0.25 implies negligible covariate imbalance between the two groups (15).

## 3 Results

### 3.1 Baseline characteristics of the study population

**Figure 1:**
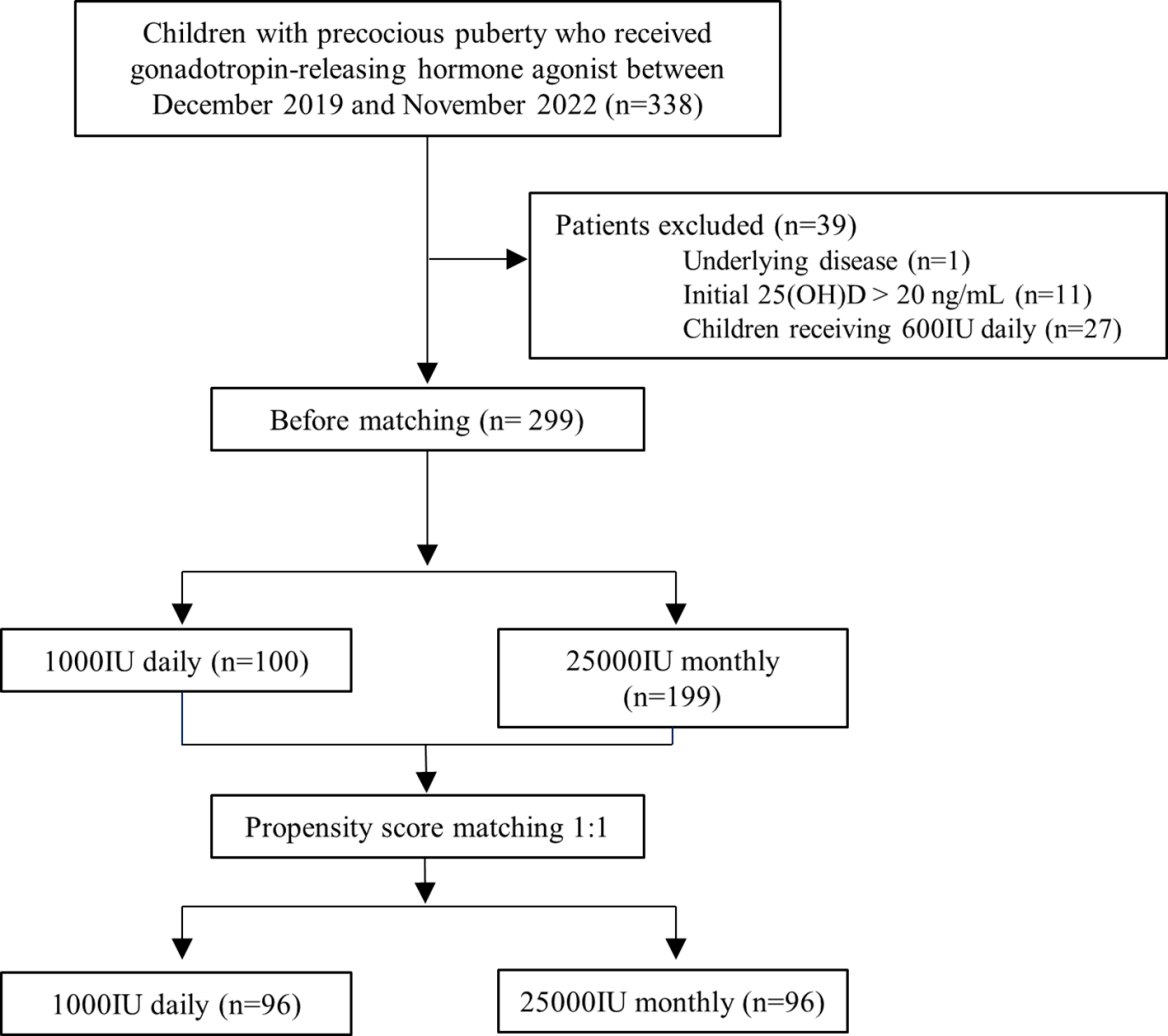
Flowchart of participants in the study. 25(OH)D, serum 25-hydroxyvitamin D.

During the study period, 388 patients were treated with GnRH agonists for precocious or advanced puberty. After excluding one patient with Graves’ disease, 11 patients with adequate 25(OH)D concentration, and 27 children who took 600 IU daily, 100 children receiving 1,000 IU of vitamin D3 every day [daily group], and 199 children receiving 25,000 IU of vitamin D3 every 4 weeks [monthly group] were included (**Figure 1**). The characteristics of the full cohort before propensity score matching are shown in **Table 1**. Compared with the monthly administration group, the daily administration group was more likely to be girls (67.0 vs 54.3%, p=0.047), had a lower height Z-score (0.6 ± 0.9 vs 0.9 ± 0.9, p=0.009), and had an initial blood collection in winter/spring rather than in summer/fall (76.0 vs 60.3%, p=0.010). Age, weight Z-score, and BMI Z-score were similar between the groups. The baseline 25(OH)D concentrations were higher in the monthly group than in the daily group (14.9 ± 3.2 vs 14.0 ± 3.1 ng/mL, p=0.014). After propensity score matching using age, sex, BMI z-score, season, and baseline 25(OH)D level, 96 patients of each daily supplement group (33 boys, age 10.5 ± 1.4 years) and monthly supplement group (33 boys, age 10.5 ± 1.3 years) were included in this study. When comparing the daily and monthly administration groups, no between-group differences were observed in height and weight Z-score. (all p > 0.05) (**Table 1**).

**Table 1:** Clinical characteristics of the study population before and after propensity score matching.

Variable	Full cohort				Propensity-score matched cohort			
	Daily group (N=100)	Monthly group (N=199)	Total (N=299)	SMD	Daily group (N=96)	Monthly group (N=96)	Total (N=192)	SMD
Age, years	10.5 ± 1.4	10.6 ± 1.4	10.5 ± 1.4	0.0153	10.5 ± 1.4	10.5 ± 1.3	10.5 ± 1.4	0.0037
Sex, *n* (%)				0.2693				0.0000
Female	67 (67.0)	108 (54.3)	175 (58.5)		63 (65.6)	63 (65.6)	126 (65.6)	
Male	33 (33.0)	91 (45.7)	124 (41.5)		33 (34.4)	33 (34.4)	66 (34.4)	
Height Z-score	0.6 ± 0.9	0.9 ± 0.9	0.8 ± 0.9		0.6 ± 0.8	0.8 ± 1.0	0.7 ± 0.9	
Weight Z-score	0.7 ± 1.1	0.9 ± 0.8	0.8 ± 0.9		0.8 ± 1.0	0.8 ± 0.8	0.8 ± 0.9	
Body mass index Z-score	0.6 ± 1.2	0.7 ± 0.9	0.7 ± 1.0	0.0698	0.7 ± 1.2	0.6 ± 0.9	0.7 ± 1.0	0.0277
Season, *n* (%)				0.3676				0.0244
Summer/fall	24 (24.0)	79 (39.7)	103 (34.4)		24 (25.0)	25 (26.0)	49 (25.5)	
Winter/spring	76 (76.0)	120 (60.3)	196 (65.6)		72 (75.0)	71 (74.0)	143 (74.5)	
Baseline 25(OH)D, ng/mL	14.0 ± 3.1	14.9 ± 3.2	14.6 ± 3.2	0.3102	14.1 ± 3.1	14.3 ± 3.3	14.2 ± 3.2	0.0667

25(OH)D, 25-hydroxyvitamin D; SMD, standardized mean difference. Continuous variables are expressed in mean ± standard deviation.

### 3.2 Comparison between the daily and the monthly groups

**Figure 2:**
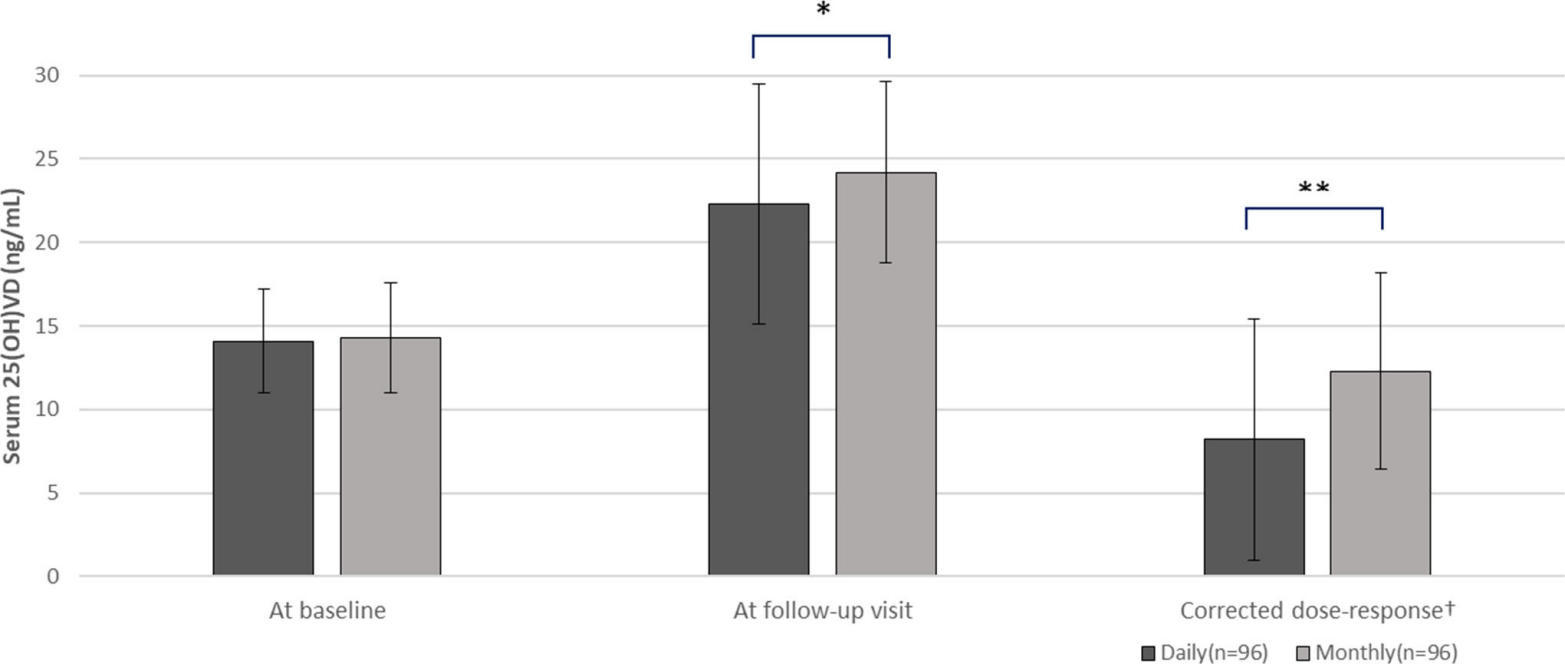
Comparison of serum 25(OH)D concentrations between daily (n=96) and monthly group (n=96). T-test comparing daily versus monthly group, *p-value <0.05, **p-value <0.01. 25(OH)D, serum 25-hydroxyvitamin D. ^†^An increasing value per 1,000 IU/daily.

We compared the serum 25(OH)D concentrations after vitamin D3 supplementation in each group (**Table 2**, **Figure 2**). Serum concentrations of 25(OH)D were similar in both groups at baseline (14.1 ± 3.1 vs. 14.3 ± 3.3 ng/mL, p = 0.65) and there was no significant difference in the time until the follow-up visit (5.8 ± 2.9 vs. 6.3 ± 1.6 months, p = 0.22). At the follow-up visit, serum 25(OH)D concentrations were significantly higher in the 25,000 IU monthly intake group than the 1,000 IU daily intake group (24.2 ± 5.4 vs. 22.3 ± 7.2 ng/mL; p = 0.037), thus the increase in serum 25(OH)D concentration was greater in monthly group (10.9 ± 5.3 vs. 8.2 ± 7.2 ng/mL; p = 0.018). Next, we evaluated the corrected dose-response by calculating the increase in serum 25(OH)D per 1,000 IU of daily vitamin D3 intake. In the monthly group, where the daily equivalent vitamin D3 intake was 892.9 IU (25,000 IU divided by 28 days), the corrected dose-response was also higher compared to the daily group (12.3 ± 5.9 vs. 8.2 ± 7.2 ng/mL increase per 1,000 IU/daily; p = 0.002).

**Table 2:** Effect and safety profile of daily and monthly administration of cholecalciferol.

	Daily group (N=96)	Monthly group (N=96)	Total (N=192)	*P-*value
Follow-up duration, months	5.8 ± 2.9	6.3 ± 1.6	5.9 ± 2.5	0.22
Serum 25(OH)D, ng/mL				
At baseline	14.1 ± 3.1	14.3 ± 3.3	14.2 ± 3.2	0.65
At follow-up visit	22.3 ± 7.2	24.2 ± 5.4	23.2 ± 6.4	0.037*
Serum 25(OH)D > 20ng/mL at follow-up visit, *n* (%)	56(58.3)	75(78.1)	131(68.2)	0.005**
Increase in serum 25(OH)D^a^, ng/mL	8.2 ± 7.2	10.9 ± 5.3	9.1 ± 6.8	0.018*
Corrected dose-response^b^ in increase in serum 25(OH)D, ng/mL per 1,000 IU	8.2 ± 7.2	12.3 ± 5.9	9.5 ± 7.1	0.002**
Serum Calcium, mg/dL				
At baseline	9.9 ± 0.3	10.0 ± 0.4	10.0 ± 0.4	0.52
At follow-up visit	10.0 ± 0.4	9.9 ± 0.5	9.9 ± 0.4	0.18

25(OH)D, serum 25-hydroxyvitamin D.

a Serum 25(OH)D concentration difference between baseline and first follow-up visit.

b An increasing value per 1,000 IU/daily.

Continuous variables are expressed in mean ± standard deviation.

The proportion of patients attaining non-deficient vitamin D status (25(OH)D level > 20 ng/mL) at the follow-up visit was higher in the monthly regimen group (n=75, 78.1%) than in the daily regimen group (n=56, 58.3%) (p = 0.005). No hypercalcemia was observed at follow-up visit in either group. (9.9 ± 0.5 vs. 10.0 ± 0.4 ng/mL).

## 4 Discussion

Our results showed that monthly administration of 25,000 IU of vitamin D3 resulted in a significantly higher increase in serum 25(OH)D concentrations than the daily dose regimen. A higher number of patients in the monthly group reached non-deficient concentrations of 25(OH)D than those in the daily group. Hypercalcemia was not observed in either the monthly or daily dose groups.

In our study, only 11 out of a total 338 patients had sufficient 25(OH)D concentrations, while the rest were deficient. The prevalence of vitamin D deficiency in children and adolescents is increasing in Korea, in line with the worldwide trend (16).

South Korea is located in the Northern Hemisphere, where it is difficult to synthesize sufficient amounts of vitamin D during the winter and spring due to the low solar altitude (17). In addition, the majority of children have limited opportunities for outdoor activities because they mostly resided in urban areas and spend most of their time in schools or academies due to the unique educational environment (18). Furthermore, a previous survey reported that the dietary intake of vitamin D3 was much lower than the DRI suggested by the Ministry of Health and Welfare in 2020 in Korean (200 IU daily for infants and children under 11 years of age and 400 IU daily for children aged 12 years and older) (19, 20). Therefore, vitamin D3 supplementation is necessary to reach adequate 25(OH)D concentrations.

Although most guidelines recommend daily vitamin D3 supplementation, adherence to the daily intake has consistently been a concern (8). As a result, intermittent high dose regimen have been explored as an alternative approach considering the long half-life of vitamin D (21) both in adults (22–25) and children (10–12). Ish-Shalom et al. (22) reported a similar efficacy of vitamin D3 supplementation with the same cumulative dose of daily, weekly, and monthly regimens after 2 months of vitamin D3 intake in elderly women who were followed up for hip fractures. Ganie et al. (23) also reported no significant difference between daily (600IU, 1,000IU and 2,000IU) and monthly (60,000IU) dosing regimen in vitamin D deficient adults until 24 weeks after the intervention. Meanwhile, Dalle et al. (24) demonstrated that a 50,000 IU monthly administration regimen is more effective in correcting hypovitaminosis D than the 1,750 IU daily administration, as well as a research by De Niet et al. (25), which proved a shorter time to reach optimal serum 25(OH)D concentrations in the monthly administration of 50,000 IU vitamin D3 group than in the daily 2,000 IU group. Although the results of the studies conducted in adults are heterogeneous, they suggest that intermittent high-dose therapy is as effective as daily dosing therapy in adult.

In children, several studies also have investigated the efficacy and safety of vitamin D3 administration at various doses and intervals. In a study by Carnes et al. (10) that compared 25(OH)D concentrations after 12 months of administration of 300,000 IU, 150,000 IU, and placebo at 6-month intervals in adolescents, only the 300,000 IU group achieved a higher 25(OH)D concentration than the placebo group. Ghazi et al. (11) compared different doses of vitamin D3 of 50,000 IU monthly, 50,000 IU bimonthly, and placebo in high-school adolescents residing in Taleghan (latitude 36.51°N), and 50,000 IU monthly and bimonthly administration groups showed significantly higher 25(OH)D concentrations than the placebo group; however, these studies evaluated the effectiveness of an intermittent high dosing schedule compared to placebo, rather than compared to daily dosing. There has been only one study comparing the efficacy of a monthly dosing regimen with a daily dosing regimen in breastfed infants from mothers who were vitamin D deficiency (12). However, no studies have compared daily and monthly administration of vitamin D3 in healthy children and adolescents. Hence, to the best of our knowledge, our study is believed to be the first to investigate the comparison between daily and monthly administration of vitamin D3 in this specific population.

Our study demonstrates the safety of a monthly high-dose regimen and an even greater increase of serum 25(OH)D compared to daily dose administration in children, without complications of hypercalcemia. In our retrospective cohort, we specifically recruited children diagnosed with precocious puberty and vitamin D deficiency in order to mitigate the potential influence of pubertal stage on vitamin D status. The result showed that the intermittent monthly dose regimen group exhibited a greater increase in serum 25(OH)D concentrations compared to the daily dose regimen group, even when converted to a daily dose. Moreover, the proportion of individuals with 25(OH)D concentrations of > 20 ng/mL was higher in the monthly regimen group. This result may be attributed to the higher medication adherence in the monthly supplementation group, as they received vitamin D3 concomitantly with GnRH agonist injection. Our study suggests that a monthly administration of high-dose vitamin D3 is sufficient to maintain adequate serum 25(OH)D concentrations owing to the long half-life of vitamin D, while minimizing the risk of hypercalcemia.

Our study had several limitations. First, we did not fully investigate values related to calcium metabolism, such as ionized calcium, intact parathyroid hormone, 1,25-dihydroxyvitamin D3, or the presence of hypercalciuria. Additionally, other clinical factors that could affect vitamin D synthesis, such as sunlight exposure time and intake of natural food products or fortified foods rich in vitamin D, have not been investigated. We were also unable to determine compliance in the daily administration group because we did not accurately check for adherence to intake at each visit or ascertain the average number of days for administration. Also, the accuracy of the administered vitamin D3 dosage remained uncertain as we did not standardize the individual responsible for administering vitamin D3, whether it was the child, a parent, or a healthcare professional. As random assignment was not used, there may have been a bias towards children who seemed to have lower compliance with the monthly dose of vitamin D3. Finally, we did not consider the quantity of adipose tissue, a reservoir for vitamin D3 (26), due to a lack of data on body composition components. Although we adjusted for BMI Z-score, it may not provide a complete representation of adipose tissue content. However, the strength of our study is that we applied propensity score matching to overcome the selection bias. We compared study groups with uniform characteristics who were diagnosed with precocious or early puberty and received GnRH agonists during the study period, thus maintaining a prepubertal state. Additionally, as only patients receiving GnRH agonists every 4 weeks were included, compliance with monthly vitamin D3 supplementation was somewhat ensured. Despite the limitations of this retrospective, small-cohort study, this is the first study to investigate the effects of monthly vitamin D3 administration in healthy children and adolescents.

## 5 Conclusion

Our results suggest that monthly supplementation with 25,000 IU of vitamin D3 can be a safe and effective alternative to daily dosing for maintaining adequate 25(OH)D concentrations in children and adolescents, considering challenges related to adherence to daily vitamin D3 supplementation. Further prospective studies are required to establish an effective intermittent dosing schedule for pediatric populations.

## Data availability statement

The original contributions presented in the study are included in the article/supplementary material. Further inquiries can be directed to the corresponding author.

## Ethics statement

The studies involving humans were approved by Institutional Review Board of Hanyang University Hospital. The studies were conducted in accordance with the local legislation and institutional requirements. The ethics committee/institutional review board waived the requirement of written informed consent for participation from the participants or the participants’ legal guardians/next of kin because 1. The research presents no more than minimal risk to the participants. 2. It is ascertained that the exemption of consent will not negatively impact the rights or welfare of the research participants. 3. Obtaining consent from the research participants is practically impossible or is deemed to severely affect the validity of the research. 4. There is no reason to presume the refusal of consent from the research participants, and the risk posed to the participants is extremely low even if the consent is waived.

## Author contributions

JC: Conceptualization, Investigation, Writing – original draft, Writing – review & editing. YC: Conceptualization, Formal Analysis, Investigation, Writing – original draft, Writing – review & editing. SY: Conceptualization, Funding acquisition, Investigation, Project administration, Supervision, Writing – review & editing.
